# Patient satisfaction and associated factor at red cross pharmacies in Addis Ababa, Ethiopia

**DOI:** 10.1186/s12913-023-10042-4

**Published:** 2023-10-30

**Authors:** Fikreselam Habte, Melak Gedamu, Chalelgn Kassaw

**Affiliations:** Department of Pharmacology and Clinical Pharmacy, School of Pharmacy, College of Health Science, Addis Ababa University, 9082 Addis Ababa, Ethiopia

**Keywords:** Patient Satisfaction, Community Pharmacy, Red Cross Pharmacies

## Abstract

**Background:**

Patient satisfaction is a crucial aspect of healthcare, reflecting the positive feelings patients experience when using a service. It serves as an indicator of the gap between expected and actual service quality from the patient's perspective. Measuring patient satisfaction is recommended for healthcare providers at all levels as it contributes to improvement efforts**.** In recent times, pharmacy services have evolved beyond merely supplying medications to becoming more patient-centered and caring**.** Given the high number of patients relying on the limited Red Cross community pharmacies in the city, this study aims to assess patient satisfaction and identify factors associated with patient satisfaction towards Red Cross Pharmacies in Addis Ababa, Ethiopia.

**Patients and methods:**

Cross sectional study design was conducted from August 15 to August 30, 2022 in three Red Cross Pharmacies in Addis Ababa. Patients were selected by Convenience sampling technique. Structured questionnaire was used to assess patient satisfaction. Bivariate and Multivariate logistic regression were computed to assess statistical association between the outcome variable, and independent variables. SPSS version 21 was used for analysis.

**Results:**

Four hundred seven participants were willing and completed the study. The overall satisfaction towards Red Cross pharmacy service was 60.4%. Inadequate counselling was main reason for dissatisfaction (45%). Regarding associated factors, unavailability of some medications (Adjusted odds ratio = 0.393, 95% CI: 0.208–0.741), unfair medication cost (Adjusted odds ratio = 0.613, 95% CI: 0.607–0.910), and lack of organized pharmacy work flow (Adjusted odds ratio = 0.105, 95% CI: 0.049–0.221) were negatively associated with clients’ satisfaction.

**Conclusion:**

This study provides significant insights into patient satisfaction with Red Cross pharmacy services in Addis Ababa, Ethiopia, revealing an overall patient satisfaction rate of 60.4%. While a substantial number of patients had positive experiences, dissatisfaction due to inadequate counseling was a notable concern. Factors negatively associated with patient satisfaction, including medication unavailability, unfair cost, and a lack of organized workflow, further highlight the need for targeted interventions to improve patient experiences. Addressing these issues will be critical to enhance pharmaceutical care services and bridge the gap between patient needs and satisfaction.

## Introduction

Patient satisfaction is the degree of positive feeling that patients experience having used a service. It is one of important aspect of health care. It indicates the gap between expected service and the actual experience from the patient point of view [1]. Routine measuring patient satisfaction level for improvement is the recommended approach for health care provider at all level. Also, measurement of patient satisfaction has shown to be an increasingly important determination of overall health outcomes [2]. It has been observed in many studies that patients who are satisfied with health service are adherent to their treatment, enjoy better health, and there was more efficient use of health care resource [3].

Recently pharmacy service has been increasingly expanded beyond simple medication supply to become a more patient-centered and caring service. Pharmacists work in harmony with other health professionals to achieve the best clinical outcome and to improve patients' quality of life. The pharmacist is also expected to have an appropriate caring attitude and to apply his/her pharmacotherapy knowledge and skill for the improvement of patients' health and well-being [4, 5].

Patient-centered approach of pharmacy service is expected to increase patient satisfaction. In Ethiopia patient centered clinical pharmacy service is mostly implemented in public hospitals. However, such approached are not implemented adequately in community pharmacies. Still community pharmacy is considered as medication selling area only, without due emphasis on patient care. Despite this, development of Good Dispensing Practice (GDP) manual by Ethiopian Food and Drug Authority (EFDA) may have core value for pharmaceutical care service, ultimately increasing patient satisfaction. The issue is there is no clear monitoring whether GDP manual is adequately implemented or not [6].

Worldwide, various studies have been performed to assess patient satisfaction with community Pharmacies service [7–11].While this measure becomes a key marker in developed countries, interest in the evaluation of patient satisfaction is growing in developing countries to evaluate community pharmaceutical service [12–15].

In Ethiopia, some studies had been conducted on the satisfaction of client with pharmacy service in public hospitals [18–25]. However, there is scarce documentation of opinion of patient satisfaction towards community pharmacy service. Red Cross pharmacies are the only chain pharmacies serving the patients throughout Ethiopia, serving larger segment of the population. Red cross pharmacy are known for lower price of medicines compared to private community pharmacy because of this many patients want to use such type of pharmacies, however in Addis Ababa there are only three Red Cross Pharmacies for more than five million population in the city [16]. Medicines availability, price, patients waiting times, accessibilities of the pharmacies, and services provided by the pharmacists may affect patient satisfaction [7, 17, 18].

Despite the vital role of patient satisfaction in shaping the quality of healthcare services, there is a paucity of research focusing on patient satisfaction towards community pharmacy services in Ethiopia, particularly at Red Cross pharmacies. Patient satisfaction is a significant indicator of healthcare service delivery and has been associated with improved treatment adherence, better health outcomes, and efficient utilization of healthcare resources. However, the factors affecting patient satisfaction at Red Cross pharmacies in Addis Ababa remain largely unexplored, leading to a gap in understanding the quality of pharmacy services provided to the population. The aim of this study was therefore, to assess patient satisfaction and identify factor associated with patient satisfaction toward Red Cross Pharmacies in Addis Ababa.

## Methods

### Study area, design and period

Ethiopian Red Cross society (ERCS) was established in 1935 after the second Italian aggression over Ethiopia. Addis Ababa Branch Red Cross society was established in 1973. ERCS operates Red Cross Community Pharmacies in Ethiopia. These pharmacies are part of the ERCS's efforts to provide accessible and affordable healthcare services to the public. Red Cross Community Pharmacies are known for offering medications at lower prices compared to private community pharmacies, making them an important resource for many patients.

The Red Cross Community Pharmacies aim to deliver quality pharmaceutical services to the community, focusing on patient-centered care and promoting health outcomes. They collaborate with other healthcare professionals to ensure the best clinical outcomes for patients and contribute to improving the overall quality of life of the population. Red Cross pharmacy serves 500 customers within a day. Currently Addis Ababa has an estimated population of around 5, 22,800. The study was conducted in the three Red Cross Pharmacies found in Addis Ababa. The locations of the pharmacies are in center of the city that many people have chance to visit those pharmacies.

The cross-sectional study was conducted between August 15 and 30, 2022, aimed to collect data on patient satisfaction and associated factors at Red Cross pharmacies in Addis Ababa during that specific time frame. The findings from such a study can help inform the current state of patient satisfaction in the community and identify potential areas for improvement in the pharmacy services provided by Red Cross pharmacies.

### Study population

All patients visiting Red Cross Pharmacies were source population. Among the source population patients’ visiting Red Cross pharmacies during the data collection period and fulfill eligibility criteria were sample population.

Eligibility Criteria for this study were; all patients who were purchasing medications for themselves and age greater than18 years. Patient who were mentally unstable; ill to respond; buying non-pharmaceutical products and supplies and peoples purchasing medications for others were excluded from the study.

### Study variable

Dependant variable was patient satisfaction. Independent variable includes: Socio –demographic characteristic (age, sex, education status, occupation, family income, residence); payment mode for medicines; Pharmacy service; pharmacy setting.

### Sampling techniques

All participants were selected by convenience sampling method. Patients were interviewed at exit from pharmacy until the required number of patients for each day was achieved.

The sample size was determined by a formula for estimating single population proportion assuming a confidence level of 95% and 10% non-response rate were added. From study conducted in Yekatit hospital, Addis Ababa, patient satisfaction was 51.6%.

The formula was:

$$n=\frac{{\left(\frac{Z\alpha }{2}\right)}^{2}XP\left(1-P\right)}{{D}^{2}}$$

where-

*n* = is the required sample size.

*P* = 51.6%, is from patient satisfaction proportion prevalence study in Yekatit hospital to get maximum number [19].

D = margin of error tolerated which is 5%

Zα/2 = 1.96 at 95% confidence level (α of 5%).

10% were added for non- response rate accordingly, the required calculated s

$$n=\frac{{\left(1.96\right)}^{2}X 0.51\left(1-0.51\right)}{{0.05}^{2}}\approx 379$$

When 10% added, the sample size was; $n = 379 + 37.9 = 416.9 \approx 417$

After proportionally allocating patient number for each pharmacy (417/3 ~ 139) Around 140 patients were interviewed from each pharmacy. The data was collected for 15 days. In each date around 10 patients were interviewed from each pharmacy.

### Data collection and analysis

Data collection was conducted by face to face interview using structured questionnaire. The questionnaire was developed by reviewing different literatures related to the topic done in Ethiopia [19–23]. The questioner was initially prepared in English and then translates into Amharic and back to English. Three pharmacists who speak Amharic were hired for the data collection. The data collectors were trained for one day on procedures, techniques and ways of collecting the data.

The questioner was pre-tested in private community pharmacies on around 5% of the study participant. Twenty-one patients were involved for the pre-test. Data were checked for consistency and completeness. Principal Investigator had the responsibilities to supervise the data collectors and to check whether the questionnaire was complete or not.

After collecting and thoroughly cleaning the data, the next step was to conduct analyses using SPSS v.22 software. The purpose of these analyses was to examine various variables and relationships within the dataset, with a focus on understanding patient satisfaction and associated factors at Red Cross pharmacies in Addis Ababa. The analyses could yield valuable insights into the pharmacy services' performance, patient experiences, and potential areas for improvement. Descriptive statistics was conducted to get a summary of key variables, such as patient demographics (age, gender, etc.), patient satisfaction scores, and other relevant variables. The analysis was also involved calculating the average patient satisfaction score and identifying the proportion of patients who were satisfied, neutral, or dissatisfied with the pharmacy services. Regression analysis was conducted to identify factors associated with patient satisfaction.

## Results

### Socio-demographic characteristics of study participants

From a total sample size of 417 participants, 407 patients were included in the final analysis with a response rate of 97.6%. Most of the study participants were male (53.6%); married (55.0%), and were in the age group 29–39 years (39.5%). Around one third (24.6%) of the study participants were employed in governmental institutions, and lived in urban areas (80.1%). Most of the patients purchase medications out of pocket (84.5%), the credit payment is to denote for those who pay for their medication through their employer company (Table 1).

**Table 1:** Socio-demographic characteristics of the study participants at Red Cross pharmacy, Addis Ababa, Ethiopia, 2022

Variable	Category	Frequency (%)
**Sex**	Male	218 (53.6)
	Female	189 (46.4)
**Age**	18–28	144 (35.4)
	29–39	160 (39.5)
	40–49	82 (20.1)
	≥ 50	21 (5.2)
**Marital status**	Single	149 (36.6)
	Married	224 (55.5)
	Divorced	34 (8.4)
**Occupation**	Government employee	100 (24.6)
	Unemployed	54 (13.3)
	Merchant	63 (15.5)
	Daily labourers	102 (25.0)
	Student	88 (21.6)
**Current residence**	Urban	326 (80.1)
	Rural	81 (19.9)
**Payment mode**	In cash	344 (84.5)
	Credit	63 (15.5)

### Patients’ satisfaction with Red Cross Pharmacy

The overall satisfaction with Red Cross Pharmacy was 60.4%. Among them 83.8% of patients were satisfied with the pharmacy facility setup. Around 82.3% of the patients were satisfied with the cleanliness of the pharmacy and its waiting area (Table 2).

**Table 2:** The satisfaction status of clients at Red Cross pharmacy, Addis Ababa, Ethiopia, 2022

Variables	Satisfied	Neutral	Dissatisfied
	Frequency (%)	Frequency (%)	Frequency N (%)
**1 Satisfaction with Pharmacy setup**			
Location of the pharmacy is comfortable	381(93.6%)	15(3.6%)	10(2.4%)
Pharmacy location related with other health service	298(73.2%)	19(4.5%)	90(22.1%)
Waiting area is clean and comfortable	335(82.3%)	36(8.8%)	36(8.8%)
Cleanness of pharmacy	335(82.3%)	51(12.5%)	21(5.2%)
Convenience of dispensing area	357(87.7%)	13(3.2%)	37(9.1%)
**2 Pharmacist-patient interaction**			
Willingness to answer question	322(79.1%)	16(3.9%)	69(16.9%)
Pharmacist easy and understandable language	314(77.1%)	12(2.9%)	81(19.9%)
Respect and politeness	274(67.3%)	31(7.6%)	102(25.1%)
Service waiting time	216(53.1%)	22(5.4%)	169(41.5%)
Spends enough counselling time	153(37.6%)	9(2.2%)	245(60.2%)
**3 Pharmacist counselling on**			
Medication dosage regimen	333(81.9%)	6(1.4%)	68(15.7%)
Possible side effects	130(31.9%)	8(2.0%)	269(66.1%)
Medication interactions	65(15.9%)	2(.5%)	340(83.6%)
Storage of medicines	125(30.7%)	1(.4%)	281(69.0%)
Labelling on dispensed drugs	263(64.6%)	7(1.7%)	113(33.7%)
**4 Medicine availability and cost**			
Prescribed drugs availability	153(37.6%)	231(56.8%)	23(5.6%)
Cost of the medication	125(30.7%)	3(0.7%)	279(68.6%)

Around 62.8% of the patients were satisfied with pharmacist communication and interaction with patient. Many patients were dissatisfied with short counselling time (60.2%) and long waiting time to get pharmaceutical service (41.5%). Otherwise, more than two-third of the sampled clients were satisfied by pharmacists’ respect, politeness, easy and understandable language usage and answering to their queries (Table 2).

Less than half of the patients (45%) were satisfied by counseling provided by the pharmacists. The major reasons for dissatisfaction were poor counseling on side effect of the medication; interaction of the drug with other drug, food and drinks; and how to store the drug in home. Otherwise, most patients (81.9%) were satisfied by the counseling on dosage regimen of the medication (administration route, dose of the medicine, frequency of administration and duration of treatment). Most patients (68.6%) were dissatisfied by the cost of medication (Table 2).

### Factors associated with patient satisfaction towards Red Cross pharmacy

In the bivariate logistic regression analysis residence; payment type; availability of the drug on the day of pharmacy visit; patients’ belief on cost of medication; pharmacy location, and organization of workflow in the pharmacy showed significant associated with satisfaction. After incorporating those associated variables in to multivariate logistic regression only availability of medication, cost of medication and organization of work flow in the pharmacy had significant association with patient satisfaction. Patients who don’t get their full prescribed medication were 60% (AOR = 0.393, 95% CI: 0.208–0.741, P = 0.003) less likely to be satisfied compared to those patients who have gotten all prescribed drugs. Moreover, patients who thought that price of prescribed medications were 0.613 times (AOR = 0.613, 95% CI: 0.607–0.910, P = 0.024) more likely to be satisfied compared to clients who thought that price of prescribed medications was not fair (Table 3).

**Table 3:** Multivariable logistic regression analysis of factors associated with client satisfaction at Red Cross Pharmacy, Addis Ababa, Ethiopia, 2022

Variables		Satisfied N (%)	Dissatisfied N (%)	COR (95% CI)	AOR (95% CI)	P-value
Current residence	Urban	118(36.2)	208(63.8)	**1**	**1**	
	Rural	47(58.0)	34(42.0)	0.41(0.25,0.674)^*^	0.45(0.19,1.078)	0.073
Medication availability on day of interview	All medication available	40(26.1)	113(45.9)	**1**	**1**	
	Some medication available	120(49.8)	134(50.2)	0.350(0.219,0.560)	0.393(0.208,0.741)	0.003
Cost of medication	Fair price	45(36.0)	80(64.0)	**1**	**1**	
	Not fair price	80(28.1)	160(71.3)	0.756(0.489,1.169)	0.613(0.607,0.910)	0.024
Pharmacy location	Good	140(37.8)	230(62.2)	**1**	**1**	
	Neutral	4(28.6)	10(71.4)	0.166(0.046,0.605)	0.105(0.022,0.511)	0.057
	Not Good	7(30.4)	16(69.6)	0.391(0.165,0.928)	0.362(0.124,1.057)	0.063
Organization of Pharmacy work flow	Good	90(30.8)	202(69.2)	**1**	**1**	
	Neutral	21(46.7)	24(53.3)	0.509(0.270,0.962)	0.598(0.264,1.35)	0.204
	Not good	54(77.1)	16(22.9)	0.132(.072,0.243)	0.105(0.049,0.221)	0.0001

## Discussion

Patient satisfaction with Red Cross pharmacy services being found to be 60.4% suggests that a significant portion of the patients who utilized Red Cross pharmacies in the study expressed satisfaction with the services they received. This finding is an essential indicator of the quality of healthcare services delivered by Red Cross pharmacies and reflects how well they meet the needs and expectations of their patient population. The study reveals that the majority of patients had a positive experience with Red Cross pharmacy services, indicating that the pharmacies are successful in delivering satisfactory healthcare experiences. While 60.4% is a considerable proportion of satisfied patients, it also indicates that there is room for improvement. The satisfaction level was better than most pharmacy service of hospitals in Ethiopian [23–26]. It was also better than some other African countries pharmacies, Tanzania (46%) [20]. But lower than developed countries satisfaction level, which was 65% in United Arab Emirates [7],85.1% in USA [3] and 77%in Qatar [27].

This study showed that patient satisfaction in Red Cross Community Pharmacy was higher than patient satisfaction in most hospitals’ pharmacy in Ethiopia. Possible reasons contributing to a better satisfaction rate at Red Cross pharmacies compared to other pharmacies include their reputation as a humanitarian organization, affordable medication prices, caring and empathetic staff, patient-centered approach, community trust, ethical practices, adherence to GDP, collaboration with public hospitals, and active participation in healthcare initiatives. These factors may collectively contribute to a positive patient experience, fostering higher satisfaction levels among patients visiting Red Cross pharmacies in Addis Ababa.

In this study, patients’ satisfaction with the pharmacy setup was 83.8%. This is higher than patient satisfaction in most public hospital in Ethiopia [19, 21–23] and other countries [15]^.^ The pharmacy's working area setup plays a crucial role in achieving a patient satisfaction rate. An organized and efficient working area design enables streamlined medication dispensing, reduces waiting times, and ensures clear communication between patients and pharmacy staff. The pharmacy's layout, with easy access to medication shelves, consultation areas, and prescription counters, enhances the patient experience, making it convenient and comfortable for patients to access healthcare services. A well-arranged working area also fosters a patient-centered environment, where staff can offer personalized care and address patient needs promptly. The implication of this high satisfaction rate is a positive reputation for the pharmacy, increased patient loyalty, and improved health outcomes as satisfied patients are more likely to adhere to medications and follow treatment plans.

EFDA takes strict follow-up and measures on community pharmacy setup more than public hospitals pharmacies, that could be also the reason for the decreased patient satisfaction with public hospital pharmacy building. Also, there is no clear criterion on the number of pharmacies that should be available in public hospitals that depends on number of patients served. Many patients are served in a single outpatient pharmacy without having enough waiting area. This also could be reason for dissatisfaction in public hospital pharmacy [23, 24, 26]. It is tire some when a diseased man is required to stand for long period of time without adequate waiting area.

Patient satisfaction with interaction with the pharmacist in this study was, 62.8%. This is also better than patient satisfaction in some public hospital pharmacies in Ethiopia [21, 26].But it was lower than other public hospital in Ethiopia [21, 23, 25]. The reason could be patients may rate differently on different days about their interaction with the pharmacist. Relatively patients in public hospitals pharmacies in Ethiopia had better satisfaction on interaction with the patient than patients in Red Cross pharmacy. This could be because patients using public hospitals are relatively poor and easily satisfied with minor things relatively to patients in community pharmacy.

Main reason for decreased patient satisfaction in Red Cross community pharmacy is related with counseling. Only 45% of patients were satisfied with counseling service. Pharmacists in community pharmacy focus on telling the patient about the dosage regimen of the medication, they give little attention on counseling about the side effect, drug interaction, precautions and storage of the medication. Most community pharmacies are private owned and majorly focus on selling the medication in a shortest time possible without letting adequate time on detail counseling. Also, there is no additional payment for counseling service that could be the reason pharmacists give little attention on counseling in community pharmacies. Public hospitals in Ethiopia had comparable or better counseling than community pharmacy [19, 21, 22].There was some difference in satisfaction with the specific counseling point.

Additionally, clinical pharmacy service and pharmaceutical care programs are initiated in only in public hospitals. This program should be implemented in every day counseling activities in community pharmacies. Since this program may help to optimize medication use in patients. But until now there is no strict rule to initiate such programs in community pharmacies. Until now EFDA doesn’t have any strict follow-up about counseling sessions. Furthermore, patients do not inquire information about medications they take about its side effect, interactions and precautions. Patients focus on the dosage regimen of the medicine only. That could be the possible reason for decreased patient satisfaction in Red Cross pharmacy compared to public hospital pharmacies in Ethiopia. Counseling on possible side effect, drug interaction and storage of medication is main reason for dissatisfaction in public hospital also [19, 21, 23, 24]. This indicate there should be reminder for major counseling points in every dispensing area.

Different factors were associated with clients’ satisfaction in this study. Disorganized flow of work in the pharmacy, unavailability of all prescribed medicines, and higher medication cost were negatively associated with patient satisfaction. In public hospital pharmacy there is program called Auditable Pharmaceutical Transaction System (APTS). In APTS there is clear steps for patient flow starting from entering the pharmacy and evaluation of the prescription until the patient leave the pharmacy [28]. But APTS is not practiced in Red Cross community pharmacy. This could be reason decreased patient satisfaction in Red Cross pharmacy. It will be easier for patient if there is clear step that patients should follow when entering Red Cross pharmacies.

Availability of only some medications from the all prescribed medication was also negatively associated with clients’ satisfaction. Mostly patients want to purchase all of their prescribed medication at one place, but due to unavailability only some medication may be available in one pharmacy, this is commonly seen in community pharmacies. It is common to see patients worried about where to find the unavailable medication. This is also the case with other studies [13, 15].

Recently due to COVID-19 and political instability in Ethiopia cost of medication has raised alarmingly. Many patients purchase medications from out of pocket, health insurance system is initiated recently for health expenses. This is also the main reason for dissatisfaction for patients in Ethiopia. On the other hand, unlike public hospital pharmacies, Red Cross pharmacy purchase medications from private medication suppliers. That could be also the reason for patient dissatisfaction with the price of medication. This is also the case with other studies done in Pakistan where free or lower cost medication increased patient satisfaction [15].Unlike the present study, different studies showed that age, gender, marital status, level of education were associated with clients’ satisfaction [15, 29, 30]. The difference might be in part due to difference in studied facilities.

## Conclusion

Measuring the level of patient satisfaction using various dimensions of services helps to predict the gap between patient needs and patient satisfaction regarding pharmaceutical care services. This study provides valuable insights into patient satisfaction with Red Cross pharmacy services in Addis Ababa, Ethiopia. The overall patient satisfaction rate of 60.4% highlights that a more than half of patients had positive experiences with the pharmacy services. However, there is room for improvement as nearly half of the dissatisfied patients attributed their dissatisfaction to inadequate counseling. Factors negatively associated with patient satisfaction include unavailability of some medications, unfair medication cost, and a lack of organized pharmacy workflow. These findings emphasize the need for targeted interventions to address these issues and enhance patient experiences in Red Cross pharmacies.

Based on the study findings, we recommend implementing targeted interventions to enhance patient satisfaction and pharmaceutical care services at Red Cross pharmacies in Addis Ababa, Ethiopia. Efforts should focus on improving counseling services to address patient dissatisfaction, ensuring the availability of medications, and reviewing medication pricing practices for fairness and transparency. Additionally, optimizing the pharmacy workflow and fostering a patient-centered approach will help create a more organized and patient-friendly environment. Continuous monitoring and evaluation of patient satisfaction levels will be crucial to gauge the impact of these interventions and drive ongoing improvements in pharmacy services, ultimately enhancing patient experiences and meeting their healthcare needs effectively.

## Data Availability

The datasets used and/or analysed during the current study are available from the corresponding author on reasonable request.

## Abbreviations

AOR: Adjusted odd ratio
APTS: Auditable pharmaceutical transaction system
COR: Crude odd ratio
CI: Confidence interval
EFDA: Ethiopian food and drug authority
